# Additive and Dominance Genomic Analysis for Litter Size in Purebred and Crossbred Iberian Pigs

**DOI:** 10.3390/genes13010012

**Published:** 2021-12-22

**Authors:** Houssemeddine Srihi, José Luis Noguera, Victoria Topayan, Melani Martín de Hijas, Noelia Ibañez-Escriche, Joaquim Casellas, Marta Vázquez-Gómez, María Martínez-Castillero, Juan Pablo Rosas, Luis Varona

**Affiliations:** 1 Departamento de Anatomía, Embriología y Genética Animal, Facultad de Veterinaria, Instituto Agrolimentario de Aragón (IA2), 50013 Zaragoza, Spain; houssemsrihi@unizar.es (H.S.); mmartinezcastillero@gmail.com (M.M.-C.); 2 Area de Producció Animal, Centre UdL-IRTA, 25198 Lleida, Spain; JoseLuis.Noguera@irta.cat; 3 Departamento de Ciència Animal, Universitat Politècnica de València, 46071 Valencia, Spain; victoriatopayan@gmail.com (V.T.); noeibes@dca.upv.es (N.I.-E.); 4 Departament de Ciència Animal i dels Aliments, Universitat Autònoma de Barcelona, 08193 Barcelona, Spain; melani.mhv@gmail.com (M.M.d.H.); joaquim.casellas@uab.cat (J.C.); Marta.Vazquez@uab.cat (M.V.-G.); 5 Programa de Mejora Genética “Castúa”, INGA FOOD S.A. (Nutreco), Avda. A Rúa, 2—Bajo Edificio San Marcos, 06200 Almendralejo, Spain; juan.rosas@nutreco.com

**Keywords:** pig, Iberian, additive, dominance, genetic correlation, crossbreeding, genomic selection

## Abstract

INGA FOOD S. A., as a Spanish company that produces and commercializes fattened pigs, has produced a hybrid Iberian sow called CASTÚA by crossing the Retinto and Entrepelado varieties. The selection of the parental populations is based on selection criteria calculated from purebred information, under the assumption that the genetic correlation between purebred and crossbred performance is high; however, these correlations can be less than one because of a GxE interaction or the presence of non-additive genetic effects. This study estimated the additive and dominance variances of the purebred and crossbred populations for litter size, and calculated the additive genetic correlations between the purebred and crossbred performances. The dataset consisted of 2030 litters from the Entrepelado population, 1977 litters from the Retinto population, and 1958 litters from the crossbred population. The individuals were genotyped with a GeneSeek^®^ GGP Porcine70K HDchip. The model of analysis was a ‘biological’ multivariate mixed model that included additive and dominance SNP effects. The estimates of the additive genotypic variance for the total number born (TNB) were 0.248, 0.282 and 0.546 for the Entrepelado, Retinto and Crossbred populations, respectively. The estimates of the dominance genotypic variances were 0.177, 0.172 and 0.262 for the Entrepelado, Retinto and Crossbred populations. The results for the number born alive (NBA) were similar. The genetic correlations between the purebred and crossbred performance for TNB and NBA—between the brackets—were 0.663 in the Entrepelado and 0.881 in Retinto poplulations. After backsolving to obtain estimates of the SNP effects, the additive genetic variance associated with genomic regions containing 30 SNPs was estimated, and we identified four genomic regions that each explained > 2% of the additive genetic variance in chromosomes (SSC) 6, 8 and 12: one region in SSC6, two regions in SSC8, and one region in SSC12.

## 1. Introduction

The Iberian pig breed is one of the porcine populations that has the highest meat quality [1]. Historically, Iberian pig production was developed extensively with purebred varieties, which took advantage of the *Dehesa* environment in southwestern Spain. In recent decades, however, many traditional production systems have been substituted with intensive production systems that use crossbreeding with Duroc populations to improve growth and efficiency [2]. The norms that regulate Iberian pig production [3] obligate farmers crossing Iberian and Duroc varieties to cross boars from the Duroc variety and sows from the Iberian variety. Prolificacy, which is lower than that of white pig populations, is the major limitation in the intensive production of crossbred pigs from Iberian dams [4]. The INGA FOOD, S.A. company has developed a crossbreeding scheme between two Iberian varieties (Retinto–R- and Entrepelado–E-) that has created a hybrid sow called CASTUA–ER, which has an important heterosis effect in prolificacy [5]. In addition, the company has been developing a breeding scheme for increasing litter size through selection in the parental Retinto and Entrepelado populations.

Theoretically, the optimal strategy for the selection of purebreds for crossbred performance is Recurrent Reciprocal Selection [6]; however, it has not been routinely used in pig breeding because it involves a delay in the generation interval. In fact, purebred parental populations are selected based on selection criteria calculated from purebred phenotypic information, and under the assumption that the genetic correlation between purebred and crossbred performance is high [7]. Those genetic correlations can be imperfect (<1) because of genotype-by-environment (GxE) interactions and the presence of non-additive genetic effects [7].

Genomic information facilitates the analysis of crossbreeding data, even if genotyped and phenotyped individuals are not directly related [8], by the definition of an additive-dominance genotypic model that provides estimates of genotype x environmental interactions through genotypic correlations. In addition, the estimates of genotypic and dominance variances can be used to estimate the additive genetic correlation between purebred and crossbred performances. Backsolving, as proposed by Wang et al. [9], provides an estimate of the SNP effects and allows us to calculate the amount of additive genetic variance associated with each genomic region in purebred and crossbred performances.

This study estimated the additive and dominance genotypic variances and covariances, which were used to calculate the additive and dominance genetic variances and the genetic correlations between purebred and crossbred performances in the Retinto and Entrepelado populations. In addition, the distribution of the additive genetic variance within the autosomal genome for purebred and crossbred performance was quantified.

## 2. Materials and Methods

The phenotypic data included the number of piglets born alive (NBA) and the total number born (TNB) for 306 Entrepelado and 313 Retinto purebred sows, and for 333 crossbred (Entrepelado x Retinto) sows when crossed with Entrepelado, Retinto or Duroc boars (Table 1).

All of the sows were genotyped with the GeneSeek^®^ GPP Porcine 70K HDchip (Illumina Inc., San Diego, CA, USA). Filtering excluded genotypes that had a minor allele frequency < 0.05 and an SNP call rate < 0.90 in the overall population. From that, 34,316 SNP markers were used to build the genomic relationship matrices with our own developed software in the R environment [10]. The missing genotypes were replaced with their expectation.

The model of analysis assumed that the phenotypic values of individuals (***y***) (TNB and NBA) are explained by the (biological) additive (***u***) and dominance (***v***) effects of the SNPs, and a covariate (*c*) with the average homozygosity (***f***), the systematic effects (***b***) order of parity (1, 2, 3, and >3), the sire of service breed (Entreplado, Retinto, or Duroc) and herd-year-season (122 levels). Phenotypic data were generated in three herds, and herd-year-season effects were defined every 3 months. The sow permanent environmental effects (***s***) with 2030, 1958 and 1977 levels for the Entrepelado, Retinto and Crossbred populations, and the residuals (***e***), were as follows:

$$\left[\begin{array}{c} y_{E}\\ y_{R}\\ y_{ER} \end{array}\right]=\left[\begin{array}{c} f_{E}c_{E}\\ f_{R}c_{R}\\ f_{RE}c_{RE} \end{array}\right]+\left[\begin{array}{ccc} X_{E} & 0 & 0\\ 0 & X_{R} & 0\\ 0 & 0 & X_{RE} \end{array}\right]\left[\begin{array}{c} b_{E}\\ b_{R}\\ b_{ER} \end{array}\right]+\left[\begin{array}{ccc} T_{E} & 0 & 0\\ 0 & T_{R} & 0\\ 0 & 0 & T_{RE} \end{array}\right]\left[\begin{array}{c} s_{E}\\ s_{R}\\ s_{ER} \end{array}\right]+\left[\begin{array}{ccc} T_{E} & 0 & 0\\ 0 & T_{R} & 0\\ 0 & 0 & T_{RE} \end{array}\right]\left[\begin{array}{c} u_{E}\\ u_{R}\\ u_{ER} \end{array}\right]\;+\left[\begin{array}{ccc} T_{E} & 0 & 0\\ 0 & T_{R} & 0\\ 0 & 0 & T_{RE} \end{array}\right]\left[\begin{array}{c} v_{E}\\ v_{R}\\ v_{ER} \end{array}\right]+\left[\begin{array}{c} e_{E}\\ e_{R}\\ e_{ER} \end{array}\right]$$

where ***X*** and ***T*** are the corresponding incidence matrices. Following Vitezica et al. [8], ***u*** and ***v*** can be described in terms of the vectors of additive (***a***) and dominance (***d***) SNP genotypic effects as follows:

$$\left[\begin{array}{c} u_{E}\\ u_{R}\\ u_{RE} \end{array}\right]=\left[\begin{array}{c} Za_{E}\\ Za_{R}\\ Za_{ER} \end{array}\right]\;\mathrm{and}\;\left[\begin{array}{c} v_{E}\\ v_{R}\\ v_{RE} \end{array}\right]=\left[\begin{array}{c} Wd_{E}\\ Wd_{R}\\ Wd_{ER} \end{array}\right]$$

The matrices ***Z*** = (***z_1_……z_m_***) and ***W*** = (***w_1_…….w_m_***) are equal to 1, 0, −1 and 0, 1, 0 for SNP genotypes A_1_A_1_, A_1_A_2_ and A_2_A_2_, respectively.

The covariance across individual genotypic additive (***u***) and dominance (***v***) effects is

$$cov\left[\begin{array}{c} u_{E}\\ u_{R}\\ u_{RE} \end{array}\right]=G_{o}⨂G\;\mathrm{and}\;cov\left[\begin{array}{c} v_{E}\\ v_{R}\\ v_{RE} \end{array}\right]=D_{o}⨂D$$

with

$$G_{o}=\left[\begin{array}{ccc} \sigma_{U_{E}}^{2} & \sigma_{U_{E}U_{R}} & \sigma_{U_{E}U_{ER}}\\ \sigma_{U_{E}U_{R}} & \sigma_{U_{R}}^{2} & \sigma_{U_{R}U_{ER}}\\ \sigma_{U_{E}U_{ER}} & \sigma_{U_{R}U_{ER}} & \sigma_{U_{ER}}^{2} \end{array}\right]\;\mathrm{and}\;D_{o}=\left[\begin{array}{ccc} \sigma_{V_{E}}^{2} & \sigma_{V_{E}V_{R}} & \sigma_{V_{E}V_{ER}}\\ \sigma_{V_{E}V_{R}} & \sigma_{V_{R}}^{2} & \sigma_{V_{R}V_{ER}}\\ \sigma_{V_{E}V_{ER}} & \sigma_{V_{R}V_{ER}} & \sigma_{V_{ER}}^{2} \end{array}\right]$$

and

$$G=\frac{{ZZ}^{\prime }}{\left\{tr\left[{ZZ}^{\prime }\right]/n\right\}}\;\mathrm{and}\;D=\frac{{WW}^{\prime }}{\left\{tr\left[{WW}^{\prime }\right]/n\right\}}$$

The variance components were estimated by REML [11] through the EM-REML algorithm using *remlf90* software [12] and, in order to obtain the average information matrix, we used one extra iteration with *airemlf90*. Additive and dominance variance components were calculated in each of the populations (*E*, *R*, and *ER*) as follows:

(1) $$\left[\begin{array}{c} {\hat{\sigma }}_{aE}^{2}\\ {\hat{\sigma }}_{aR}^{2}\\ {\hat{\sigma }}_{aER}^{2} \end{array}\right]=\left[\begin{array}{c} \frac{{\hat{\sigma }}_{U_{E}}^{2}}{\left\{tr\left[ZZ^{\prime }\right]/n\right\}}\\ \frac{{\hat{\sigma }}_{U_{R}}^{2}}{\left\{tr\left[ZZ^{\prime }\right]/n\right\}}\\ \frac{{\hat{\sigma }}_{U_{ER}}^{2}}{\begin{array}{c} \left\{tr\left[ZZ^{\prime }\right]/n\right\} \end{array}} \end{array}\right]\;\;\;\;\;\mathrm{and}\;\;\;\;\;\left[\begin{array}{c} {\hat{\sigma }}_{dE}^{2}\\ {\hat{\sigma }}_{dR}^{2}\\ {\hat{\sigma }}_{dER}^{2} \end{array}\right]=\left[\begin{array}{c} \frac{{\hat{\sigma }}_{D_{E}}^{2}}{\left\{tr\left[WW^{\prime }\right]/n\right\}}\\ \frac{{\hat{\sigma }}_{D_{R}}^{2}}{\left\{tr\left[WW^{\prime }\right]/n\right\}}\\ \frac{{\hat{\sigma }}_{D_{ER}}^{2}}{\begin{array}{c} \left\{tr\left[WW^{\prime }\right]/n\right\} \end{array}} \end{array}\right]$$

The additive ($\sigma_{A}^{2})$ and dominance ($\sigma_{D}^{2})$ genetic variances of the purebred populations were calculated as follows:

(2) $${\hat{\sigma }}_{A_{E}}^{2}=\sum_{i=1}^{n}2{\hat{p}}_{Ei}{\hat{q}}_{Ei}{\hat{\sigma }}_{a_{E}}^{2}+2{\hat{p}}_{Ei}{\hat{q}}_{Ei}{\left({\hat{q}}_{Ei}-{\hat{p}}_{Ei}\right)}^{2}{\hat{\sigma }}_{d_{E}}^{2}$$

(3) $${\hat{\sigma }}_{D_{E}}^{2}=\sum_{i=1}^{n}{\left(2{\hat{p}}_{Ei}{\hat{q}}_{Ei}\right)}^{2}{\hat{\sigma }}_{d_{E}}^{2}$$

(4) $${\hat{\sigma }}_{A_{R}}^{2}=\sum_{i=1}^{n}2{\hat{p}}_{Ri}{\hat{q}}_{Ri}{\hat{\sigma }}_{a_{R}}^{2}+2{\hat{p}}_{Ri}{\hat{q}}_{Ri}{\left({\hat{q}}_{Ri}-{\hat{p}}_{Ri}\right)}^{2}{\hat{\sigma }}_{d_{R}}^{2}$$

(5) $${\hat{\sigma }}_{D_{R}}^{2}=\sum_{i=1}^{n}{\left(2{\hat{p}}_{Ri}{\hat{q}}_{Ri}\right)}^{2}{\hat{\sigma }}_{d_{R}}^{2}$$

where ${\hat{p}}_{Xi}$ and ${\hat{q}}_{Xi}$ are the raw estimates of the allelic frequencies for A_1_ and A_2_ at the *ith* SNP marker and the *X* = {*E*,*R* or *ER*} population, respectively. The estimates of the contributions to the additive variance in the crossbred population from the Entrepelado ($\sigma_{A_{ER\left(E\right)}}^{2}$) and Retinto ($\sigma_{A_{ER\left(R\right)}}^{2}$) were obtained by [8] as follows:

(6) $${\hat{\sigma }}_{A_{ER\left(E\right)}}^{2}=\sum_{i=1}^{n}2{\hat{p}}_{Ri}{\hat{q}}_{Ri}{\hat{\sigma }}_{a_{ER}}^{2}+2{\hat{p}}_{Ri}{\hat{q}}_{Ri}{\left({\hat{q}}_{Ei}-{\hat{p}}_{Ei}\right)}^{2}{\hat{\sigma }}_{d_{ER}}^{2}$$

(7) $${\hat{\sigma }}_{A_{ER\left(R\right)}}^{2}=\sum_{i=1}^{n}2{\hat{p}}_{Ei}{\hat{q}}_{Ei}{\hat{\sigma }}_{a_{ER}}^{2}+2{\hat{p}}_{Ei}{\hat{q}}_{Ei}{\left({\hat{q}}_{Ri}-{\hat{p}}_{Ri}\right)}^{2}{\hat{\sigma }}_{d_{ER}}^{2}$$

Following Vitezica et al. [8], the additive variance in the crossbred population was the average of the two values resulting from Equations (6) and (7), as follows:

(8) $${\hat{\sigma }}_{A_{ER}}^{2}=\frac{1}{2}{\hat{\sigma }}_{A_{ER\left(E\right)}}^{2}+\frac{1}{2}{\hat{\sigma }}_{A_{ER\left(R\right)}}^{2}$$

The estimate of the dominance variance of the crossbred population [8] was calculated as follows:

(9) $${\hat{\sigma }}_{D_{ER}}^{2}=\sum_{i=1}^{n}4{\hat{p}}_{Ei}{\hat{q}}_{Ei}{\hat{p}}_{Ri}{\hat{q}}_{Ri}{\hat{\sigma }}_{d_{ER}}^{2}$$

With these estimates, the heritabilities ($h_{X}^{2})$ and dominance ratios ($d_{X}^{2})$ in the purebred (*X = E*,*R*) and crossbred (*X = ER*) populations were obtained by:

(10) $${\hat{h}}_{X}^{2}={\hat{\sigma }}_{A_{X}}^{2}/\left({\hat{\sigma }}_{A_{X}}^{2}+{\hat{\sigma }}_{D_{X}}^{2}+{\hat{\sigma }}_{S_{X}}^{2}+{\hat{\sigma }}_{E_{X}}^{2}\right)$$

(11) $${\hat{d}}_{X}^{2}={\hat{\sigma }}_{D_{X}}^{2}/\left({\hat{\sigma }}_{A_{X}}^{2}+{\hat{\sigma }}_{D_{X}}^{2}+{\hat{\sigma }}_{S_{X}}^{2}+{\hat{\sigma }}_{E_{X}}^{2}\right)$$

where ${\hat{\sigma }}_{S_{X}}^{2}$ and ${\hat{\sigma }}_{E_{X}}^{2}$ are the estimates of the sow permanent environmental and residual variance in the *X = {E*,*R*,*ER}* population.

The covariance between purebred and crossbred additive genetic effects in the Entrepelado ($\sigma_{A_{E}A_{ER\left(E\right)}}$) and Retinto ($\sigma_{A_{R}A_{ER\left(R\right)}}$) populations were as follows:

(12) $${\hat{\sigma }}_{A_{E}A_{ER\left(E\right)}}=\sum_{i=1}^{n}2{\hat{p}}_{Ei}{\hat{q}}_{Ei}{\hat{\sigma }}_{a_{E}a_{ER}}+2{\hat{p}}_{Ei}{\hat{q}}_{Ei}\left({\hat{q}}_{Ei}-{\hat{p}}_{Ei}\right)\left({\hat{q}}_{Ri}-{\hat{p}}_{Ri}\right){\hat{\sigma }}_{d_{E}d_{ER}}$$

(13) $${\hat{\sigma }}_{A_{E}A_{ER\left(R\right)}}=\sum_{i=1}^{n}2{\hat{p}}_{Ri}{\hat{q}}_{Ri}{\hat{\sigma }}_{a_{R}a_{ER}}+2{\hat{p}}_{Ri}{\hat{q}}_{Ri}\left({\hat{q}}_{Ri}-{\hat{p}}_{Ri}\right)\left({\hat{q}}_{Ei}-{\hat{p}}_{Ei}\right){\hat{\sigma }}_{d_{R}d_{ER}}$$

with

(14) $$\left[\begin{array}{c} {\hat{\sigma }}_{a_{E}a_{ER}}\\ {\hat{\sigma }}_{a_{R}a_{ER}} \end{array}\right]=\left[\begin{array}{c} \frac{{\hat{\sigma }}_{U_{E}U_{ER}}}{\left\{tr\left[ZZ^{\prime }\right]/n\right\}}\\ \frac{{\hat{\sigma }}_{U_{R}U_{ER}}}{\left\{tr\left[ZZ^{\prime }\right]/n\right\}} \end{array}\right]\;\;\mathrm{and}\;\;\left[\begin{array}{c} {\hat{\sigma }}_{d_{E}d_{ER}}\\ {\hat{\sigma }}_{d_{R}d_{ER}} \end{array}\right]=\left[\begin{array}{c} \frac{{\hat{\sigma }}_{V_{E}V_{ER}}}{\left\{tr\left[WW^{\prime }\right]/n\right\}}\\ \frac{{\hat{\sigma }}_{V_{R}V_{ER}}}{\left\{tr\left[WW^{\prime }\right]/n\right\}} \end{array}\right]$$

Therefore, the genetic correlations between the purebred and crossbreed breeding values in the Entrepelado and Retinto populations were computed as follows:

(15) $${\hat{r}}_{A_{E}A_{ER\left(E\right)}}=\frac{{\hat{\sigma }}_{A_{E}A_{ER\left(E\right)}}}{\sqrt{{\hat{\sigma }}_{A_{E}}^{2}{\hat{\sigma }}_{A_{ER\left(E\right)}}^{2}}}\;\mathrm{and}\;{\hat{r}}_{A_{R}A_{ER\left(R\right)}}=\frac{{\hat{\sigma }}_{A_{R}A_{ER\left(R\right)}}}{\sqrt{{\hat{\sigma }}_{A_{R}}^{2}{\hat{\sigma }}_{A_{ER\left(R\right)}}^{2}}}$$

The vector of the SNP additive effects (${\hat{a}}_{E}$, ${\hat{a}}_{R}$ and ${\hat{a}}_{ER}$) was obtained by backsolving [9], as

(16) $${\hat{a}}_{E}=\frac{{\hat{\sigma }}_{a_{E}}^{2}}{{\hat{\sigma }}_{U_{E}}^{2}}ZG^{-1}{\hat{u}}_{E},\;{\hat{a}}_{R}=\frac{{\hat{\sigma }}_{a_{R}}^{2}}{{\hat{\sigma }}_{U_{R}}^{2}}ZG^{-1}{\hat{u}}_{R}\;\mathrm{and}\;{\hat{a}}_{ER}=\frac{{\hat{\sigma }}_{a_{ER}}^{2}}{{\hat{\sigma }}_{U_{ER}}^{2}}ZG^{-1}{\hat{u}}_{ER}$$

and the vector of the SNP dominance effects (${\hat{d}}_{E}$, ${\hat{d}}_{R}$ and ${\hat{d}}_{ER}$) was as follows:

(17) $${\hat{d}}_{E}=\frac{{\hat{\sigma }}_{d_{E}}^{2}}{{\hat{\sigma }}_{V_{E}}^{2}}WD^{-1}{\hat{v}}_{E},\;{\hat{d}}_{R}=\frac{{\hat{\sigma }}_{d_{R}}^{2}}{{\hat{\sigma }}_{V_{R}}^{2}}WD^{-1}{\hat{v}}_{R}\;\mathrm{and}\;{\hat{d}}_{ER}=\frac{{\hat{\sigma }}_{d_{ER}}^{2}}{{\hat{\sigma }}_{V_{ER}}^{2}}WD^{-1}{\hat{v}}_{ER}$$

With those, the genetic additive variances ($\sigma_{A_{E}\left(k\right)}^{2},\sigma_{A_{R}\left(k\right)}^{2},\sigma_{A_{ER\left(E\right)}\left(k\right)}^{2}\;\mathrm{and}\;\sigma_{A_{ER\left(R\right)}\left(k\right)}^{2}$) explained by the *k*th segment of the genome were calculated as follows:

(18) $${\hat{\sigma }}_{A_{E}\left(k\right)}^{2}=\sum_{i=1}^{n\left(k\right)}2{\hat{p}}_{Ei}{\hat{q}}_{Ei}{\hat{a}}_{E_{i}}^{2}+2{\hat{p}}_{Ei}{\hat{q}}_{Ei}{\left({\hat{q}}_{Ei}-{\hat{p}}_{Ei}\right)}^{2}{\hat{d}}_{E_{i}}^{2}$$

(19) $${\hat{\sigma }}_{A_{R}\left(k\right)}^{2}=\sum_{i=1}^{n\left(k\right)}2{\hat{p}}_{Ri}{\hat{q}}_{Ri}{\hat{a}}_{R_{i}}^{2}+2{\hat{p}}_{Ri}{\hat{q}}_{Ri}{\left({\hat{q}}_{Ri}-{\hat{p}}_{Ri}\right)}^{2}{\hat{d}}_{R_{i}}^{2}$$

(20) $${\hat{\sigma }}_{A_{ER\left(E\right)}\left(k\right)}^{2}=\sum_{i=1}^{n\left(k\right)}2{\hat{p}}_{Ri}{\hat{q}}_{Ri}{\hat{a}}_{ER_{i}}^{2}+2{\hat{p}}_{Ri}{\hat{q}}_{Ri}{\left({\hat{q}}_{Ei}-{\hat{p}}_{Ei}\right)}^{2}{\hat{d}}_{ER_{i}}^{2}$$

(21) $${\hat{\sigma }}_{A_{ER\left(R\right)}\left(k\right)}^{2}=\sum_{i=1}^{n\left(k\right)}2{\hat{p}}_{Ei}{\hat{q}}_{Ei}{\hat{a}}_{ER_{i}}^{2}+2{\hat{p}}_{Ei}{\hat{q}}_{Ei}{\left({\hat{q}}_{Ri}-{\hat{p}}_{Ri}\right)}^{2}{\hat{d}}_{ER_{i}}^{2}$$

where *n*(*k*) is the number of SNP markers within the kth segment, which was set to 30 after testing several number of the SNP markers (20, 30 and 40). In order to identify the genes within the genomic regions that explained >2.0% of the total genetic variance, we used the biomart tool (www.ensembl.org (accessed on 10 October 2021)).

## 3. Results and Discussion

The results based on TNB and NBA were similar, which was expected because these two traits have a high genetic correlation [13], and the raw correlation between them in the analyzed dataset was 0.94; therefore, we focused on the results with the TNB, and the results for NBA are presented as Supplementary Information (Tables S1–S3 and Figure S1). The REML estimates of the additive genotypic (co) variances are shown in Table 2 and Table 3 in TNB and NBA.

The additive genotypic variance was higher in the crossbred populations than it was in the purebred populations. This may be due to scale effects, as the phenotypic variation in also greater. In addition, the estimates of the genotypic covariances between purebreds (Entrepelado and Retinto) and the crossbred population were all high and positive, and they corresponded to additive genotypic correlations of 0.704 ($0.259/\sqrt{0.248\times 0.546}$) between Entrepelado and Crossbred pigs, 0.988 ($0.388/\sqrt{0.546\times 0.282}$) between Retinto and Crossbred pigs, and 0.756 (0.200/$\sqrt{0.248\times 0.282})$ between the two purebreds. These results indicated that the genotype x environmental interaction was small, and the additive genotypic correlations were similar to those obtained by Vitezica et al. [8] in white pig populations. The REML estimates of the dominance genotypic (co)variances ranged from 0.170 (Retinto) to 0.265 (Crossbred) (Table 3).

The estimates of the dominance genotypic covariances were all positive, and reflected genotypic dominance correlations >0.95. The analysis provided the REML estimates of the sow permanent and residual effects (Table 4). The residual variance ($\sigma_{E}^{2})$ is greater in the crossbred population than in purebreds, consistently with the greater phenotypic variation. In contrast, the estimate of the sow environmental variance ($\sigma_{R}^{2})\;$ was very low in the crossbred population.

The additive and dominance genotypic (co) variances were used to calculate the additive and dominance genetic variances in the purebred populations based on expressions (1) to (5) (Table 3). The estimates of the additive genetic variances were 0.170 (Entrepelado) and 0.150 (Retinto), and the estimates of the dominance genetic variances were 0.074 (Entrepelado) and 0.056 (Retinto). The heritability estimates were calculated using Equation (10); they were 0.052 (Entrepelado) and 0.037 (Retinto), which were within the range or slightly lower than those of white pigs [13,14,15] and in the same [5] or other Iberian [16,17] populations. The dominance ratios were obtained from Equation (11), and were 0.023 for Entrepelado and 0.014 for Retinto. They were smaller than the heritabilities, but their ratios with them were approximately 40%, which was higher than those reported for white pig populations [8,18] for litter size and similar to the results of Tusell et al. [19] in other swine traits.

We used Equations (6) and (7) to calculate the additive variances for crossbred performance in the purebred populations, which were 0.413 (Entrepelado) and 0.293 (Retinto). Therefore, the additive genetic variance in the crossbred population was the average of the two (0.353), which was higher than the additive genetic variances in the purebred populations, which were similar to the results of Vitezica et al. [8] with regard to litter size, and to the results of Tusell et al. [19] for other pig traits. Nevertheless, Xiang et al. [20] found the opposite in a cross between Landrace and Yorkshire breeds (0.86 and 0.54 in purebreds and 0.28 in crossbreds). In the present study, the dominance genetic variance in the crossbred population (0.079) was calculated based on the Equation (8), which was similar to the dominance genetic variance in the purebreds; however, its ratio with the additive genetic variances was lower (22%). Given those variance components, the heritability and dominance ratio estimates in the crossbred population were 0.072 and 0.016, respectively.

In addition, the additive genetic correlations between purebred and crossbred performances in the Entrepelado and Retinto populations were calculated based on expressions (12) to (15), which were 0.663 in Entrepelado and 0.881 in Retinto populations. Those correlations were within the range of the estimates summarized by Wientjes and Calus [7], and suggest that the efficiency of the selection for increased crossbred performance by selecting for purebred performance will be more effective in Retinto than in Entrepelado pigs.

**Figure 1:**
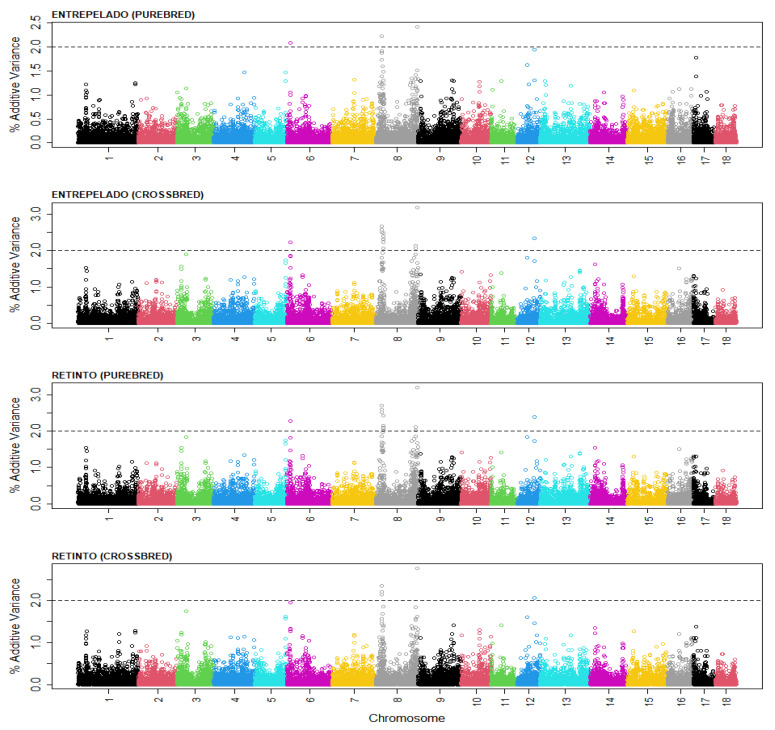
Distribution of the percentage of the additive genetic variance explained by genomic segments of 30 SNPs within the autosomal genome of purebred and crossbred performance for the total number born (TNB) in the Entrepelado and Retinto varieties. Black: chromosomes 1, 9 and 17; red: chromosomes 2, 10 and 18; green: chromosomes 3 and 11; deep blue: chromosomes 4 and 12; blue: chromosomes 5 and 13; purple: chromosomes 6 and 14; yellow: chromosomes 7 and 15; grey: chromosomes 8 and 16.

We used Equations (16) and (17) to calculate the additive and dominance genotypic effects associated with each of the 34,316 SNP markers, which were used in Equations (18)–(21) to calculate the proportion of the additive genetic variance that was explained by segments of 30 consecutive SNPs (Figure 1). The distribution of the additive variance explained by segments of 20 and 40 SNP markers were similar, and are presented as supplementary information (Figures S2 and S3).

The figure presents the distribution of the additive variance along the autosomal chromosomes in the Entrepelado and Retinto populations, and for the purebred and crossbred performance. Four genomic regions can be highlighted; each explained >2% of the additive genetic variance in at least one of the populations. The SNPs at the center of each of the genomic regions that explained the highest amount of additive genetic variance, and the genes in the Sus_Scrofa 11.1. genomic map that were within 1 Mb downstream or upstream, are presented in Table 5.

Among those genes, several can be proposed as candidate genes to explain the additive genetic variation. The genomic region surrounding bp 7,597,405 in SSC6 included BCO1 (*β-Carotene Oxygenase 1*), which encodes an enzyme that catalyzes the breakdown of provitamin A and provides retinoids for embryogenesis [21,22]. Furthermore, the GCSH (*Glycine Cleavage System H*) protein plays an important role in embryonic viability [23].

Two genomic regions were identified in SSC8 around bp 11,585,865 and bp 137,540,516. Among the genes within those regions, PRDM8 (*PR/SET Domain 8*) is involved in the neurogenesis [24] of the FGF5 *(Fibroblast Growth Factor 5*), a member of the fibroblast growth factor family that is involved in several biological processes, including embryonic development, cell growth, and morphogenesis [25,26].

The genomic region around bp 46,079,417 in SSC12 contains, among others, the GIT1 (*G protein-coupled receptor kinase interactor 1*) gene, which plays a role in spine morphogenesis [27], the NSRP1 (*Nuclear Speckle Splicing Regulatory Protein 1*) development process, and in utero embryonic development [28], and ANKRD1 (*Ankyrin Repeat Domain 1*), which is involved in neuron projection development [29].

The Gene Ontology (GO) terms for the biological processes for the proposed candidate genes are presented as Supplementary Table S4.

## 4. Conclusions

(1) The additive genetic variance and the heritabilities were higher in the crossbred than those in the purebred populations, (2) the genetic correlation between purebred and crossbred performances were higher in Retinto than they were in Entrepelado pigs, and (3) the additive genetic variances were heterogeneously distributed throughout the autosomal genome, and four genomic regions in SSC6, SSC8, and SSC12 with several candidate genes were identified.

## Figures and Tables

**Table 1:** Number of records (and number of sows between brackets) and the mean ± standard deviation of the number born alive and the total number born in Entrepelado, Crossbred and Retinto populations.

	Entrepelado	Crossbred	Retinto
N ^1^ (NS) ^2^	2030 (306)	1958 (333)	1977 (313)
NBA ^3^	7.75 ± 1.85	8.57 ± 2.27	8.07 ± 2.07
TNB ^4^	8.02 ± 1.89	8.80 ± 2.29	8.33 ± 2.11

^1^ N: number of records. ^2^ NS: number of sows. ^3^ NBA: number born alive. ^4^ TNB: total number born.

**Table 2:** REML estimates ± standard error (SE) of the additive genotypic (co)variances for the total number born (TNB).

	Entrepelado	Crossbred	Retinto
Entrepelado	0.248 ± 0.161	0.259 ± 0.178	0.200 ± 0.135
Crossbred	-	0.546 ± 0.268	0.388 ± 0.170
Retinto	-	-	0.282 ± 0.146

**Table 3:** REML estimates ± standard error (SE) of the dominance genotypic (co)variances for the total number born (TNB).

	Entrepelado	Crossbred	Retinto
Entrepelado	0.177 ± 0.165	0.212 ± 0.171	0.166 ± 0.152
Crossbred	-	0.262 ± 0.210	0.202 ± 0.179
Retinto	-	-	0.172 ± 0.199

**Table 4:** REML estimates ± standard error (SE) of the permanent environmental and residual variances for the total number born (TNB) in the Entrepelado, Crossbred and Retinto populations.

Variances ^1^	Entrepelado	Crossbred	Retinto
$\sigma_{S}^{2}$	0.191 ± 0.105	0.009 ± 0.029	0.268 ± 0.120
$\sigma_{E}^{2}$	2.810 ± 0.099	4.467 ± 0.155	3.534 ± 0.128

^1^ $\sigma_{S}^{2}$: Sow permanent environmental variance; $\sigma_{E}^{2}$: residual variance.

**Table 5:** SNPs at the center of each of the four genomic regions that explained > 2% of the additive genetic variance in at least one of the populations, and the genes located within 1 Mb downstream or upstream.

SNP ^1^	SSC ^2^	bp ^3^	Genes
rs326244568	6	7,597,405	*BCO1*, *PKD1L2*, *GCSH*, *ATMIN*, *CENPN*, *CDYL2*, *DYNLRB2*
rs81401202	8	11,585,865	*CD38*, *FGFBP1*, *PROM1*, *TAPT1*, *LDB2*
rs81406142	8	137,540,516	*CFAP299*, *FGF5*, *PRDM8*, *ANTXR2*
rs345468811	12	46,079,417	*TAOK1*, *ABHD15*, *TP53I13*, *GIT1*, *ANKRD1*, *CORO6*, *EFCAB5*, *NSRP1*, *SLC6A4*, *BLMH*, *TMIGD1*, *CPD*, *GOSR1*

^1^ SNP: single nucleotide polymorphism, ^2^ SSC: Sus Scrofa chromosome, ^3^ bp: base pair.

## Data Availability

The dataset used in this study will be available upon reasonable request to the corresponding author (lvarona@unizar.es).

## Supplementary Materials

The following supporting information can be downloaded at: https://www.mdpi.com/article/10.3390/genes13010012/s1. Table S1: REML estimates ± the standard error (SE) of the additive genotypic (co)variances for the number born alive (NBA). Table S2: REML estimates ± the standard error (SE) of the dominance genotypic (co)variances for the number born alive (NBA). Table S3: REML estimates ± the standard error (SE) of the permanent environmental and residual variances for the number born alive (NBA). Table S4: GO (Gene Ontology) terms for the biological process of the proposed candidate genes. Figure S1: Distribution of the percentage of the additive genetic variance explained by genomic segments of 30 SNPs within the autosomal genome of the purebred and crossbred performance for the number born alive (NBA) in the Entrepelado and Retinto varieties. Figure S2: Distribution of the percentage of the additive genetic variance explained by genomic segments of 20 SNPs within the autosomal genome of purebred and crossbred performance for the total number born (TNB) in the Entrepelado and Retinto varieties. Figure S3: Distribution of the percentage of the additive genetic variance explained by genomic segments of 40 SNPs within the autosomal genome of the purebred and crossbred performance for the total number born (TNB) in the Entrepelado and Retinto varieties.
