# Supplementary material for: Additive and Dominance Genomic Analysis for Litter Size in Purebred and Crossbred Iberian Pigs
Source: Genes (Basel). 2021 Dec 22;13(1):12. doi: 10.3390/genes13010012 (PMC8774905; doi:10.3390/genes13010012)
Supplement: Supplementary file 1 [file genes-13-00012-s001.zip › genes-1510754-supplementary.pdf]

genes

MDPI

Suplementary Material:

Additive and Dominant genomic analysis for litter size in pure and crossbred Iberian pigs.

Houssemeddine Srihi[1], José Luis Noguera[2], Victoria Topayan[3], Melani Martín de Hijas[4], Noelia Ibañez-Escriche[3], Joaquim Casellas[4], Marta Vázquez-Gómez[4], María Martínez-Castillero[1], Juan Pablo Rosas[5] and Luis Varona[1*]

Table S1. REML estimates ± standard error (SE) of the additive genotypic (co)variances for Number Born Alive (NBA).

	Entrepelado	Crossbred	Retinto
**Entrepelado**	0.147 ± 0.156	0.202 ± 0.169	0.184 ± 0.150
**Crossbred**	-	0.477 ± 0.241	0.410 ± 0.172
**Retinto**	-	-	0.359 ± 0.183

Table S2. REML estimates ± standard error (SE) of the dominance genotypic (co)variances for Number Born Alive (NBA).

	Entrepelado	Crossbred	Retinto
**Entrepelado**	0.293 ± 0.216	0.286 ± 0.182	0.204 ± 0.173
**Crossbred**	-	0.282 ± 0.188	0.202 ± 0.177
**Retinto**	-	-	0.155 ± 0.205

Table S3. REML estimates ± standard error (SE) of the permanent environmental and residual variances for Number Born Alive (NBA).

Variances[1]	Entrepelado	Crossbred	Retinto
σ_S^2	0.111 ± 0.111	0.004 ± 0.022	0.252 ± 0.126
σ_E^2	2.820 ± 0.099	4.385 ± 0.152	3.468 ± 0.125

[1] σ_S^2: Sow permanent environment variance; σ_E^2: Residual variance.

Table S4. GO (Gene Ontology) terms for biological process of the proposed candidate genes.

Gene	Name	Biological process	
BCO1	*Beta-carotene oxygenase 1*	GO:0001523<br>GO:0042574<br>GO:1901810	- retinoid metabolic process<br>- retinal metabolic process<br>- beta-carotene metabolic process
GCSH	*Glycine cleavage system protein H*	GO:0019464	- glycine decarboxylation via glycine cleavage system
ATMIN	*ATM interactor*	GO:0010628<br>GO:0044458<br>GO:0045893<br>GO:0045944<br>GO:1902857	- positive regulation of gene expression<br>- motile cilium assembly<br>- positive regulation of transcription, DNA-templated<br>- positive regulation of transcription by RNA polymerase II<br>- positive regulation of non-motile cilium assembly
CENPN	*Centromere protein N*	GO:0007059<br>GO:0051382	- chromosome segregation<br>- kinetochore assembly
DYNLRB2	*Dynein light chain roadblock-type 2*	GO:0007018	- microtubule-based movement
CD38	*CD38 molecule*	GO:0050794	- regulation of cellular process
FGFBP1	*Fibroblast growth factor binding protein 1*	GO:0090050<br>GO:1903589	- positive regulation of cell migration involved in sprouting angiogénesis<br>- positive regulation of cell migration involved in sprouting angiogenesis
PROM1	*Prominin 1*	GO:0045494<br>GO:0060042<br>GO:0060219<br>GO:0072112<br>GO:0072139<br>GO:2000768	- Photoreceptor cell maintenance<br>- retina morphogenesis in camera-type eye<br>- camera-type eye photoreceptor cell differentiation<br>- glomerular visceral epithelial cell differentiation<br>- glomerular parietal epithelial cell differentiation<br>- positive regulation of nephron tubule epithelial cell differentiation
TAPT1	*Transmembrane anterior posterior transformation 1*	GO:0045724<br>GO:0048706<br>GO:0048856	- positive regulation of cilium assembly<br>- embryonic skeletal system development<br>- anatomical structure development
LDB2	*LIM domain binding 2*	GO:0045944<br>GO:0044089<br>GO:0043549<br>GO:0035019<br>GO:0030334<br>GO:0010669<br>GO:0006357<br>GO:0001942	- positive regulation of transcription by RNA polymerase II<br>- positive regulation of cellular component biogenesis<br>- regulation of kinase activity<br>- somatic stem cell population maintenance<br>- regulation of cell migration<br>- epithelial structure maintenance<br>- regulation of transcription by RNA polymerase II<br>- hair follicle development
FGF5	*Fibroblast growth factor 5*	GO:0023019<br>GO:0010001<br>GO:0008543<br>GO:0008284	- signal transduction involved in regulation of gene expression<br>- glial cell differentiation<br>- fibroblast growth factor receptor signaling pathway<br>- positive regulation of cell population proliferation
TAOK1	*TAO kinase 1*	GO:1901985<br>GO:0097194<br>GO:0070050<br>GO:0046330<br>GO:0032874<br>GO:0016310<br>GO:0007095<br>GO:0007026<br>GO:0006974	- positive regulation of protein acetylation<br>- execution phase of apoptosis<br>- neuron cellular homeostasis<br>- positive regulation of JNK cascade<br>- positive regulation of stress-activated MAPK cascade<br>- phosphorylation<br>- mitotic G2 DNA damage checkpoint signaling<br>- negative regulation of microtubule depolymerization<br>- cellular response to DNA damage stimulus

		GO:0006468<br>GO:0000226	- protein phosphorylation<br>- microtubule cytoskeleton organization
GIT1	*GIT ArfGAP 1*	GO:2000300<br>GO:0106015<br>GO:0099171<br>GO:0090063<br>GO:0071222<br>GO:0061743<br>GO:0048666<br>GO:0048013<br>GO:0045820<br>GO:0045454<br>GO:0032691<br>GO:0032465<br>GO:0007626<br>GO:0007420<br>GO:0001957	- regulation of synaptic vesicle exocytosis<br>- negative regulation of inflammatory response to wounding<br>- presynaptic modulation of chemical synaptic transmission<br>- positive regulation of microtubule nucleation<br>- cellular response to lipopolysaccharide<br>- motor learning<br>- neuron development<br>- ephrin receptor signaling pathway<br>- negative regulation of glycolytic process<br>- cell redox homeostasis<br>- negative regulation of interleukin-1 beta production<br>- regulation of cytokinesis<br>- locomotory behavior<br>- brain development<br>- intramembranous ossification
ANKRD13B	*ankyrin repeat domain 13B*	GO:0002091	- negative regulation of receptor internalization

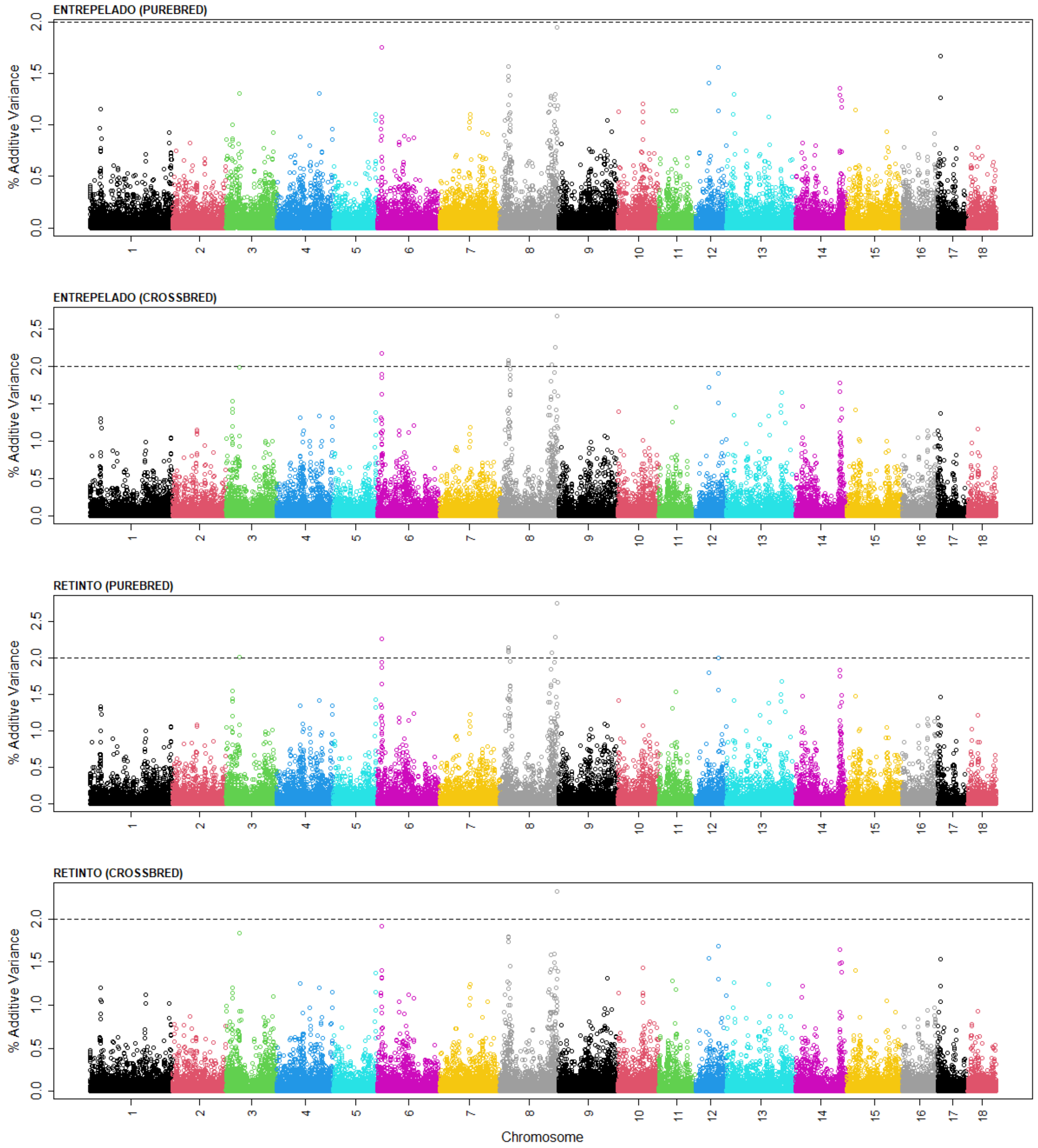

Figure S1. Distribution of the percentage of the additive genetic variance explained by genomic segments of 30 SNPs within the autosomal genome of purebred and crossbred performance for Number Born Alive (NBA) in the Entrepelado and Retinto varieties.

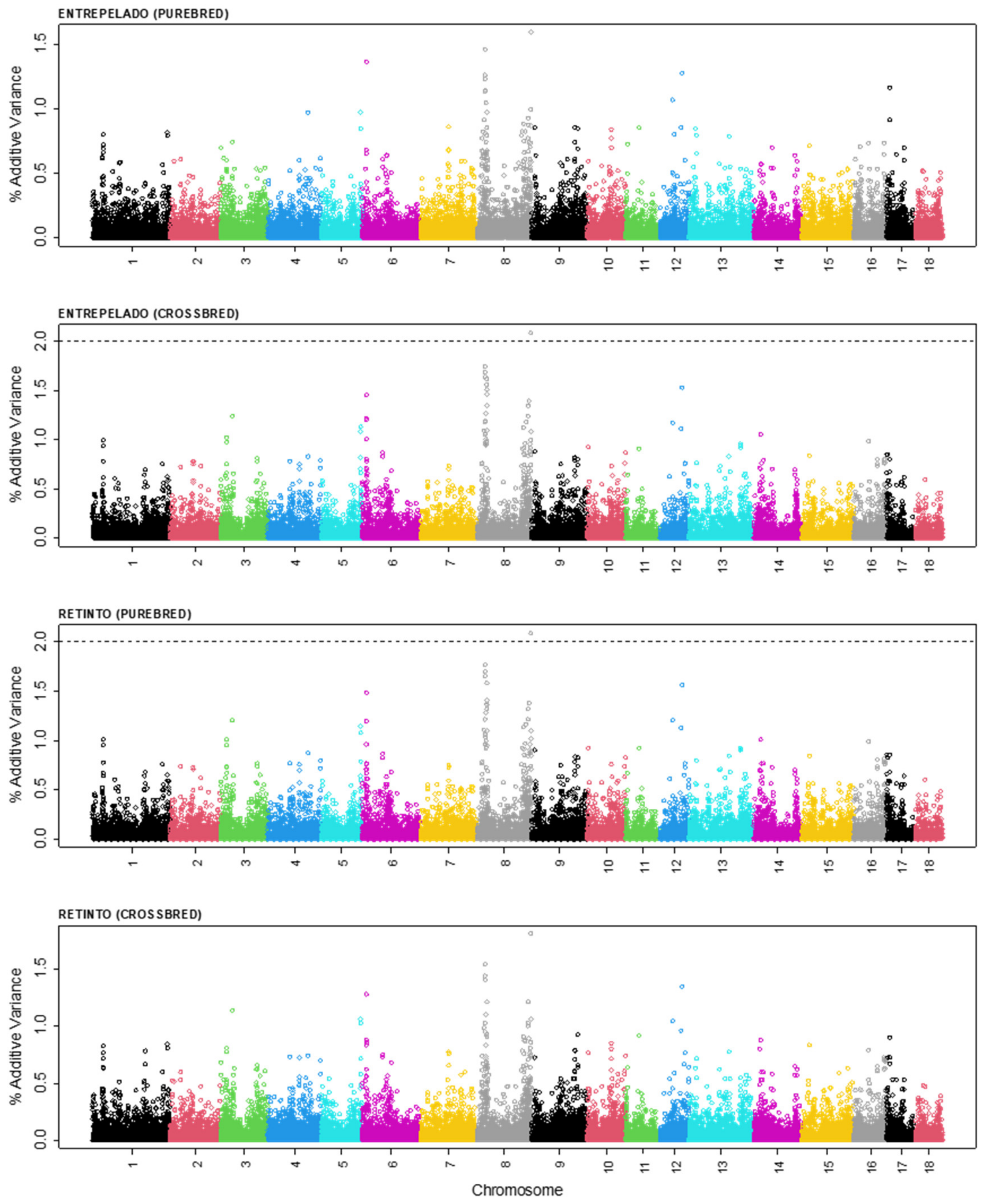

Figure S2. Distribution of the percentage of the additive genetic variance explained by genomic segments of 20 SNPs within the autosomal genome of purebred and crossbred performance for Total Number Born (TNB) in the Entrepelado and Retinto varieties.

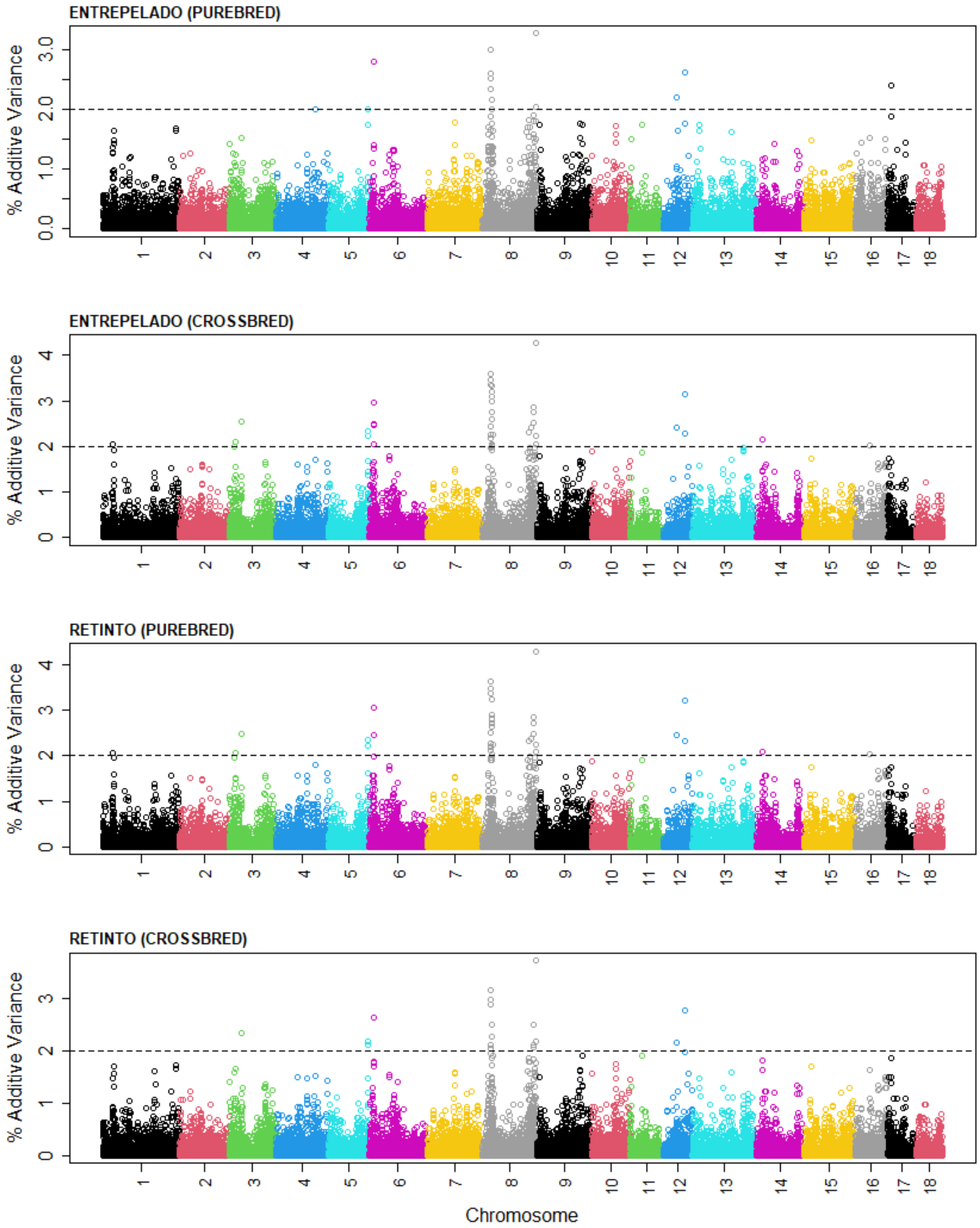

Figure S3. Distribution of the percentage of the additive genetic variance explained by genomic segments of 40 SNPs within the autosomal genome of purebred and crossbred performance for Total Number Born (TNB) in the Entrepelado and Retinto varieties.
